# Morphometric and molecular characterization of *Kudoa encrasicoli* n. sp. (Myxozoa: Myxosporea) from the European anchovy, *Engraulis encrasicolus* (L.) (Clupeiformes: Engraulidae)

**DOI:** 10.1007/s11230-022-10051-7

**Published:** 2022-07-01

**Authors:** Raúl Iglesias, Luís Filipe Rangel, Fabio Fernández-Vázquez, Maria João Santos, José M. García-Estévez

**Affiliations:** 1 Laboratorio de Parasitología, Centro de Investigación Mariña (CIM), Universidade de Vigo, Edificio de Ciencias Experimentales, Campus Lagoas-Marcosende, 36310 Vigo, Spain; 2 CIIMAR – Interdisciplinary Center of Marine and Environmental Research, Terminal de Cruzeiros do Porto de Leixões, University of Porto, Avda. General Norton de Matos s/n, 4450-208 Matosinhos, Portugal; 3 Department of Biology, Faculty of Sciences, University of Porto, FC4, Rua do Campo Alegre s/n, 4169-007 Porto, Portugal

**Keywords:** *Engraulis encrasicolus*, skeletal muscle, Myxozoa, *Kudoa*, morphometrical analysis, phylogeny

## Abstract

The European anchovy represents the main fisheries for countries in the Mediterranean and Black Sea basins. The skeletal muscle of 13 of 48 (27.1%) *Engraulis encrasicolus* (L.) specimens from North East Atlantic waters (FAO 27.8.c) was found infected with interfibrillar elongated plasmodia (130-980 µm in length) containing mature myxospores belonging to the genus *Kudoa* Meglitsch, 1947*.* No flesh softening was found associated with infection. Fresh myxospores were 10.8 ± 0.7 (9.1-12.3) µm in width 1, 11.3 ± 0.9 (9.5-13.4) µm in width 2, 6.7 ± 0.4 (5.8-7.4) µm in thickness, and 6.9 ± 0.5 (5.8-7.5) µm in length. They were almost stellate in apical view having three pointed-edged shell valves bearing three small polar capsules equal in size 5.0 ± 0.3 (4.4-5.4) μm long and 2.4 ± 0.2 (2.0-3.0) μm wide, and one rounded- to rarely bluntly pointed-edged shell valve bearing a large and particularly wide polar capsule 6.8 ± 0.4 (5.9-7.6) μm long and 4.1 ± 0.2 (3.6-4.4) μm wide. Morphological and morphometrical comparisons between these myxospores and those of *Kudoa thyrsites* (Gilchrist, 1923) from the clupeid *Sardina pilchardus* (Walbaum) (North East Atlantic waters, FAO 27.9.a)*,* with which exhibited a similarity of 98.9% and 96.2% using SSU and LSU rDNA sequences, respectively, support the creation of *Kudoa encrasicoli* **n. sp.** Morphometrical analysis of the polar capsules of flattened myxospores is suggested as a useful approach to differentiate phylogenetically related kudoids with stellate or almost stellate myxospores bearing four polar capsules.

**Supplementary Information:**

The online version contains supplementary material available at 10.1007/s11230-022-10051-7.

## Introduction

*Kudoa* Meglitsch, 1947 (Myxosporea: Multivalvulida) is the sole genus in the family Kudoidae Meglitsch, 1960, which has been traditionally characterized by having myxospores with 4 or more shell valves (SVs) and polar capsules (PCs) (Fiala et al., 2015). However, recent phylogenetic studies on the species *Kudoa eugerres* Casal, Soares, Rocha, Silva, Santos, Nascimento, Oliveira & Azevedo, 2019*, K. dicentrachi* (Sitjà-Bobadilla & Alvarez-Pellitero, 1992), and *Kudoa* sp. (from *Mugil cephalus*), which have myxospores with 2 SVs and PCs, have led to amend the genus *Kudoa* to include myxospores having 2-13 (mostly 4) SVs and PCs (Casal et al., 2019). It comprises more than 100 nominal species, most of them being histozoic myxozoans typically infecting the musculature of a large range of fish species (Moran et al., 1999; Eiras et al., 2014; Kristmundsson & Freeman 2014; Mansour et al., 2014, 2015; Shirakashi et al., 2014; Yokoyama et al., 2014; Abdel-Baki et al., 2016; Azevedo et al., 2016; Shin et al., 2016; Kasai et al., 2016a, 2016b, 2017). Some of these species (e. g. *K. thyrsites* (Gilchrist, 1923) and others) are economically important because they can form macroscopic cysts in the host’s muscle tissues and/or cause the “soft flesh”, “milky flesh” or “jelly flesh” syndrome, a postmortem myoliquefactive degeneration negatively affecting the flesh texture of certain severely infected species (Moran et al., 1999; Levsen et al., 2008; Henning & Manley 2012; Eiras et al., 2014; Marshall et al., 2016).

The genus *Engraulis* Cuvier, 1816 (Actinopterigii: Engraulidae) is composed of several commercially important species including the European anchovy, *Engraulis encrasicolus* (Linnaeus, 1758), a small pelagic fish whose distribution encompasses the eastern regions of the North and Central Atlantic Ocean, as well as the Mediterranean, Black and Azov seas (Whitehead et al., 1988). This species represents the main fisheries for the countries in the Mediterranean and Black Sea basins, where more than 300,000 tonnes were landed in 2016-2018 (FAO 2020) and it is normally commercialized fresh or processed (salted, canned - semipreserved in oil- or marinated in vinegar). Although Langdon et al., (1992) found *Kudoa* myxospores which were morphologically identified as *K. thyrsites* in the skeletal musculature of Australian (*E. australis*) and Japanese (*E. japonicus*) anchovies, no similar studies have been conducted in *E. encrasicolus* to date.

In the present paper a new *Kudoa* species infecting the musculature of the European anchovy is described based on its morphological, morphometrical and molecular characteristics.

## Materials and methods

### Fish samples and parasitological analysis

A total of 48 specimens of *E. encrasicolus* were acquired from fish markets of Vigo (Galicia, NW Spain) between May 2016 and April 2017 and transported on ice to the laboratory. The anchovies were captured by fishing vessels operating in the fishing area FAO 27.8.c (North East Atlantic-Bay of Biscay-South) which is between 43°00’ and 44°30’ N and between 2°00’ and 11°00’ W. Mean total length and weight of specimens were 13.3 ± 2.0 (10.6-17.0) cm and 18 ± 9.3 (7.4-37.1) g. Once in the laboratory, the specimens were stored at 4 °C until examination. Just before the parasitological analysis, the muscle texture of each individual was assessed subjectively by finger pressing in order to detect flesh softening associated with myoliquefaction.

For comparative purposes, 152 European pilchards (*Sardina pilchardus*) from Portuguese (n= 112; mean total length: 13.4 ± 2 cm; Aveiro and Matosinhos) and Spanish (n= 40; mean total length: 14.6 ± 3.8 cm; Rías Baixas) Atlantic waters (fishing area FAO 27.9.a - North East Atlantic-Portuguese Waters-East) were analysed for the presence of *Kudoa* myxospores, which were morphologically, morphometrically, and molecularly characterized.

The presence of myxospores in the skeletal muscles of fishes was determined by tryptic digestion according to Samaranayaka et al. (2007) with minor modifications. Briefly, three different fragments of fish musculature were excised from each fish side (one from the neck area, one from the epaxial area below the dorsal fin, and one from the lateral line area above the anal fin) and the skin was removed. A piece of each fragment weighing 100-200 mg was disposed in 10 mL of a 0.04% trypsin solution prepared by diluting a commercial 25 g/L (10x) porcine trypsin solution (Sigma-Aldrich, USA, St. Louis) in PBS pH 7.4, and then homogenized by using an IKA ULTRA-TURRAX^®^. The homogenized suspension was digested at 37 °C for 1 h and centrifuged at 2100 g and 4 °C for 15 min. The supernatant was gently aspirated, and the resulting pellet was resuspended in 500 µL of saline solution (0.15 M NaCl) and examined microscopically for the presence of myxospores. The remaining musculature of both sides of each anchovy was skinned, disposed between two transparent plastic sheets, compressed to 1-2 mm thickness by pressing with a glass plate, and examined under a stereomicroscope in order to detect plasmodia. The plasmodia-containing tissues were then extracted and used for morphometrical studies (plasmodia and myxospores) as well as histological analysis. Prevalence was calculated according to Bush et al. (1997).

### Morphometric and histological analysis

Plasmodia and myxospores were examined, measured, and photographed using an Olympus BX41 microscope (Olympus Corporation, Japan, Tokyo), a SC30 digital camera (Olympus Corporation, Japan, Tokyo) and the analySIS getIt 5.1 program (Olympus Soft Imaging Solutions GmbH, Germany, Münster). The measurements of myxospores were done on digital microphotographs taken with the 100x immersion oil objective by using the “parallel dimension” tool of CorelDRAW^®^12 (Corel Corporation, Canada, Ottawa) and a reference calibration measurement taken with the micrometer eyepiece. In addition to the measurements recommended by Burger & Adlard (2010) for describing stellate myxospores bearing unequal PCs (one PC clearly larger than the others), a second width (W1) between the edges of the two SVs bearing the large and the opposite small PCs was also measured (Giulietti et al., 2019). In the case of PCs, photographs and measurements were also taken on flattened myxospores resulting from the slight cover-glass pressure derived from the partial evaporation of saline solution. Under these conditions myxospores are immobilized and PCs are disposed in a clear longitudinal view allowing a more accurate observation and measurement. Both intact and flattened myxospores from European pilchards exhibiting myoliquefactive changes were also morphometrically studied for comparative purposes. In order to facilitate the understanding of morphometric comparative studies, the 4 PCs were named in a clockwise manner A, B, C and D (see Fig. 3), with A and C being the larger and the smaller opposite PCs, respectively, and B and D the PCs disposed in intermediate position between A and C. The ratios between the length and the width of each PC (AL/AW, BL/BW, CL/CW, and DL/DW) as well as the ratios between the lengths or the widths of the large and the small opposite PCs (AL/CL and AW/CW) were also calculated from the measurements taken on flattened myxospores of both species. In the case of *Kudoa* myxospores from *E. encrasicolus,* in which no significant differences were observed between the three small PCs, we also calculated the ratio between the length and the width by combining all data obtained for those PCs (B-C-DL/ B-C-DW).

The lengths and widths of the 4 PCs of flattened myxospores from *E. encrasicolus* and *K. thyrsites* ex *S. pilchardus* were compared using a one-way ANOVA followed by a Tukey´s multiple comparison test, after proving the normality of data by a D’Agostino & Pearson omnibus normality test. In addition, the ratios cited above excepting the B-C-DL/ B-C-DW ratio were also compared between both species using an unpaired t-test. Statistical analyses were performed using GraphPad Prism® v 5.02 (GraphPad Software, Inc.) and a level of statistical significance set to 95% (p < 0.05). A Principal Component Analysis (PCA) based on the lengths and widths of all PCs (8 variables) taken on flattened myxospores of both species was also conducted using the BioVinci software (BioTuring Inc., San Diego, CA, USA).

Some musculature fragments containing plasmodia were also fixed in 10% buffered formalin and processed by routine histological procedures. Paraffin sections (5 µm) were obtained and stained with Giemsa and haematoxylin and eosin.

### Molecular and phylogenetic analysis

Myxospore suspensions preserved in absolute ethanol from *E. encrasicolus* (isolate VKE), from *S. pilchardus* of the Spanish coast (isolate VKS) and from *S. pilchardus* of the Portuguese coast (isolate KPT) were used for the molecular analysis. Genomic DNA extraction was performed using a GenElute^TM^ Mammalian Genome DNA Miniprep Kit (Sigma-Aldrich, USA, St. Louis), following the manufacturer`s guidelines. The DNA was stored in 50 µL of TE buffer at -20 °C until further use.

Small subunit ribosomal DNA (18S rDNA) and large subunit ribosomal DNA (28S rDNA) genes were amplified by polymerase chain reaction (PCR) using universal primers as well as myxosporean-specific and kudoid-specific primers, listed in Table 1. PCRs were performed in 25 µL reactions using 0.2 µM of each primer, 0.2 mM deoxyribonucleotide triphosphates (dNTPs; NZYTech, Lisboa, Portugal), 2.5 mM MgCl_2_, 2.5 µL of 10× *Taq* polymerase buffer, 1.25 units of *Taq* DNA polymerase (NZYTech), and 2 µL (approximately 100−150 ng) of genomic DNA. The reactions were run on a Bio-Rad MJ Mini Gradient Thermal Cycler, with initial denaturation at 95 °C for 3 min, followed by 35 cycles of 94 °C for 1 min., 55 °C for 1 min., and 72 °C for 2 min. The final elongation step was performed at 72 °C for 7 min. Aliquots (5 µL) of the PCR products were electrophoresed through a 1% agarose 1× Tris acetate-EDTA buffer (TAE) gel stained with GreenSafe Premium (NZYTech). PCR products were purified and sequenced by STAB VIDA (Caparica, Portugal). The sequencing reactions were performed using a BigDye Terminator v3.1 from the Applied Biosystems Kit and were run on an ABI3730XL DNA analyser (Applied Biosystems).

**Table 1:** Sequences of forward (F) and reverse (R) PCR primers used for amplification and sequencing of *Kudoa encrasicoli* **n. sp.** and *K. thyrsites* small (18S) and large (28S) subunits of rDNA

Gene	Primer	Sequence (5’-3’)	References
18S rDNA	ERIB1 ERIB10 18e Kud6r Myxgen4r MyxospecF Kud6f Kud2f 18R	F: ACCTGGTTGATCCTGCCAG R: CTTCCGCAGGTTCACCTACGG F: CTGGTTGATCCTGCCAGT R: TCCAGTAGCTACTCATCG R: ACCTGTTATTGCCACGCT F: TTCTGCCCTATCAACTTGTTG F: TCACTATCGGAATGAACG F: TGAATGTTATAGCATGGAA R: CTACGGAAACCTTGTTACG	Barta et al. (1997) Barta et al. (1997) Hillis and Dixon (1991) Whipps et al. (2003b) Kent et al. (2000) Fiala (2006) Whipps et al. (2003b) Whipps et al. (2003b) Whipps et al. (2003a)
28S rDNA	ZX-1 1500R Kt28S1F 900F Kud2400fR 1600F L2630 U2229 L3449 Kud2400f	F: ACCCGCTGAATTTAAGCATAT R: GCTATCCTGAGGGAAACTTCG F: CAAGACTACCTGCTGAAC F: CCGTCTTGAAACACGGACCAAG R: GATGCTTGCCACTCGTAAAG F: GCAGGACGGTGGCCATGGAAG R: GGGAATCTCGTTAATCCATTCA F: TACCCATATCCGCAGCAGGTCT R: ATTCTGACTTAGAGGCGTTCA F: CTTTACGAGTGGCAAGCATC	Waeschenbach et al. (2007) Waeschenbach et al. (2007) Whipps et al. (2004) Waeschenbach et al. (2007) Shin et al. (2016) Waeschenbach et al. (2007) Waeschenbach et al. (2007) Waeschenbach et al. (2007) Lockyer et al. (2003) Grabner et al. (2012)

For the phylogenetic analysis, a set of sequences from species that consistently cluster together, according to previous studies (see Giulietti et al., 2020), into a group that constitutes a complex of *K. thyrsites* species, and other new sequences after a BLASTN search (https://blast.ncbi.nlm.nih.gov/Blast.cgi) were selected from the GenBank database. The set of selected sequences had been further filtered excluding repeated sequences, with more than 99.9 to 100% similarity to the same hosts and geographic locations and which, in many cases, were shorter in length. To make the trees more robust, some species outside this *K. thyrsites* complex group were also selected: *K. septempunctata* Matsukane, Sato, Tanaka, Kamata & Sugita-Konishi, 2010 (AB553293, AB731755) and *K. iwatai* Egusa & Shiomitsu, 1983 (AY641571, LC066366). *Unicapsula galeata* (Naidenova & Zaika, 1970) (LC474137, LC474138) was used as outgroup. Each data set contained 31 and 30 sequences for the 18S and 28S rDNA, respectively. The matrix of selected 28S rDNA sequences in GenBank was truncated corresponding to the D1-D3 regions, because most sequences in the database are only of that size.

The DNA sequences from this study and the selected data set from GenBank were aligned by multiple alignment in MAFFT v7.490 (Katoh & Standley, 2013) using the L-INS-i alignment strategy and remaining parameters as default.

For the construction of the phylogenetic trees, Maximum Likelihood (ML) was used in MEGA v7 (Kumar et al., 2016), and Bayesian Inference (BI) in MrBayes v3.2.7a (Ronquist et al., 2012). The nucleotide substitution model used, and according to the Akaike Information criteria, for both methods was the General Time Reversal with an invariable proportion of sites and a rate of changes across sites (GTR+I+G). Nodal support was calculated for ML by bootstrap resampling with 1000 replications, and for BI by the posterior probabilities estimated by Markov chain Monte Carlo (MCMC) algorithm, starting from 1,000,000 generations, with four simultaneous MCMC chains and two independent runs. The sampling frequency was 100 generations with 25% burn-in.

The consensus phylogenetic trees were edited in FigTree v1.4.4 (Rambaut, 2018) and Inkscape v1.1 (Inkscape Project, 2021).

Genetic divergence was calculated with the data sets sequences aligned for the 18S and 28S rDNA by the p-distance method (MEGA).

## Results

Kudoid myxospores and plasmodia were detected infecting the skeletal muscle of 13 of 48 specimens of *E. encrasicolus* (prevalence 27.1%)*.* No apparent flesh softening was detected in infected anchovies.

Partial 18S and 28S rDNA sequences were obtained for the new species of *Kudoa* from *E. encrasicolus* (isolate VKE: 1691 bp (GenBank accession number OM200071) and 3377 bp (OM200067), respectively), for *K. thyrsites* in *S. pilchardus* from the Spanish coast (isolate VKS: 1690 bp (OM200072) and 3421 bp (OM200068), respectively), and for *K. thyrsites* in *S. pilchardus* from the Portuguese coast (isolate KPT: 1674 bp (OM200073) and 1073 bp (OM200069), respectively). The amplification of the 28S rDNA was successful for the isolates VKE and VKS, but for the isolate KPT only the initial portion corresponding to the D1-D3 region of the 28S rDNA was amplified.

The morphological, morphometric, and molecular results presented below confirm that the plasmodia and myxospores found in the European anchovy belong to a new species of *Kudoa*.

**Family Kudoidae Meglitsch, 1960**

**Genus** ***Kudoa*** **Meglitsch, 1947**

***Kudoa encrasicoli*** **n. sp.**

*Type host: Engraulis encrasicolus* (Linnaeus, 1758), European anchovy (Clupeiformes, Engraulidae).

*Type locality:* Spanish Atlantic waters (FAO Division 27.8.c - Bay of Biscay-South).

*Site of infection:* histozoic; plasmodia located between the skeletal muscle fibres (interfibrillar).

*Prevalence:* 13 of 48 (27.1%).

*Type material:* Giemsa-stained air-dried syntype myxospores (registration number MNCN 2.05/19) and Giemsa-stained and haematoxylin and eosin-stained histological sections (registration numbers MNCN 2.05/20 and MNCN 2.05/21, respectively) are deposited in the Invertebrate Collection of Museo Nacional de Ciencias Naturales (MNCN, Madrid, Spain). A photovoucher of two intact hosts which were infected by *K. encrasicoli* **n. sp.** is also deposited in the Invertebrate Collection’s Image Archive (registration number AICI_MNCN 2.05/21).

*Etymology:* specific name comes from its host name *E. encrasicolus*.

*Representative DNA sequences:* SSU and LSU rDNA sequences were deposited in GenBank under the accession numbers OM200071 for 18S rDNA and OM200067 for 28S rDNA.

*Zoobank registration:* LSID: urn:lsid:zoobank.org:act:ED772B27-350B-447C-9270-6D3CDE397DBA.

**Fig. 1:**
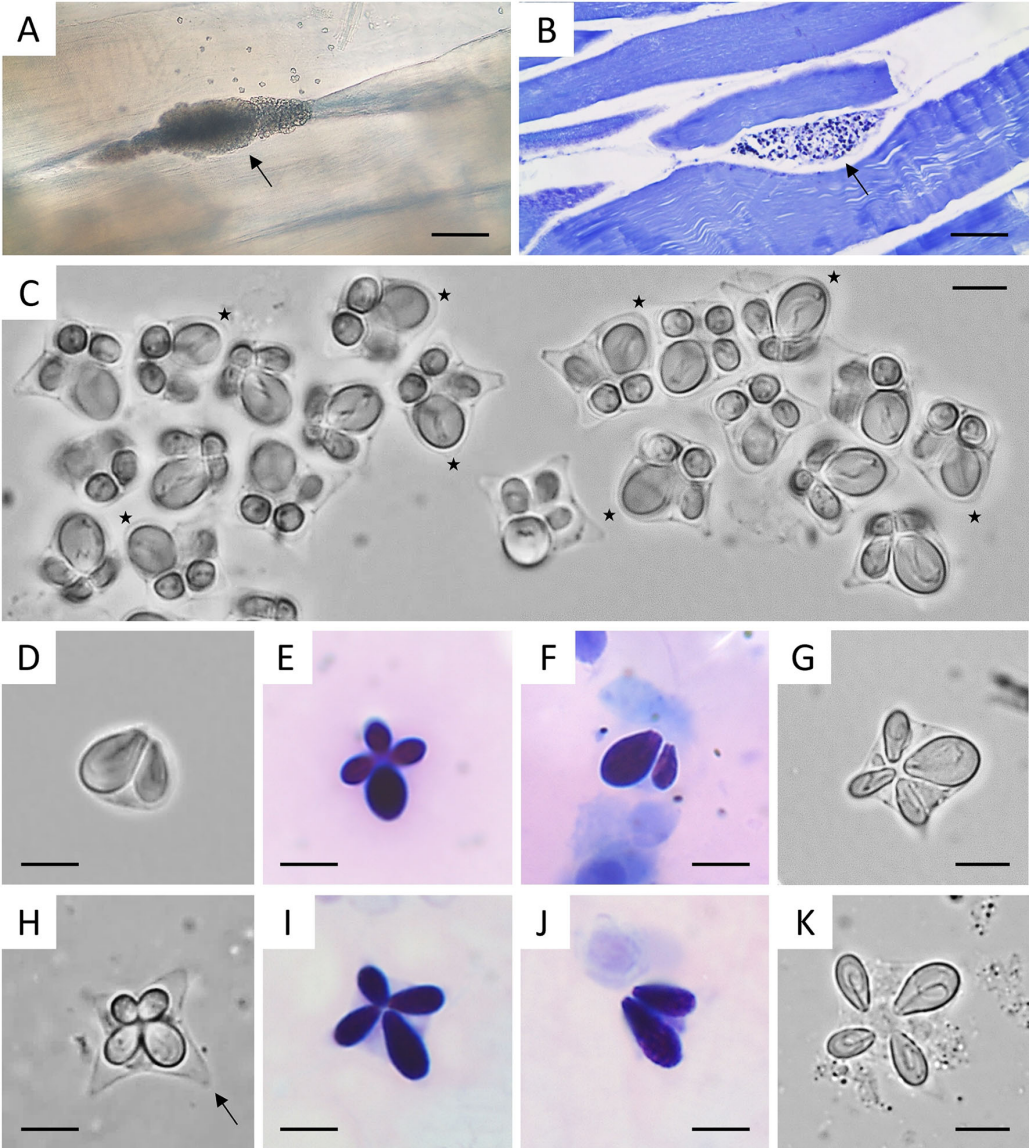
A-G. Photomicrographs of plasmodia and myxospores of *Kudoa encrasicoli* **n. sp.** from *Engraulis encrasicolus*. A-B. Interfibrillar plasmodium (arrow) observed in a wet mount (A) and in a Giemsa-stained histological section of infected skeletal muscle (B). Note in A the presence of some myxospores which are easily freed from the plasmodium because of its delicate membrane. C-D. Wet mounts of intact myxospores in apical (C) and lateral (D) view. Note the unequal PCs and the rounded to bluntly pointed edge (stars) of the SV containing the large PC. E-F. Giemsa-stained myxospores in apical (G) and lateral (H) view. G. Appearance of the four PCs in a flattened myxospore resulting from the partial evaporation of saline in the wet mount. H-K. Photomicrographs of myxospores of *K. thyrsites* from the skeletal muscle of *Sardina pilchardus* (used for morphometrical comparison with *K. encrasicoli* **n. sp.**)*.* H. Myxospore in apical view showing its typical stellate appearance with four clearly pointed SVs (arrow). I-J. Giemsa-stained myxospores in apical (K) and lateral (L) view. K. Appearance of the four PCs in a flattened myxospore resulting from the partial evaporation of saline in the wet mount. Scale bars: A-B, 100 µm; C-K, 5 µm

**Fig. 2:**
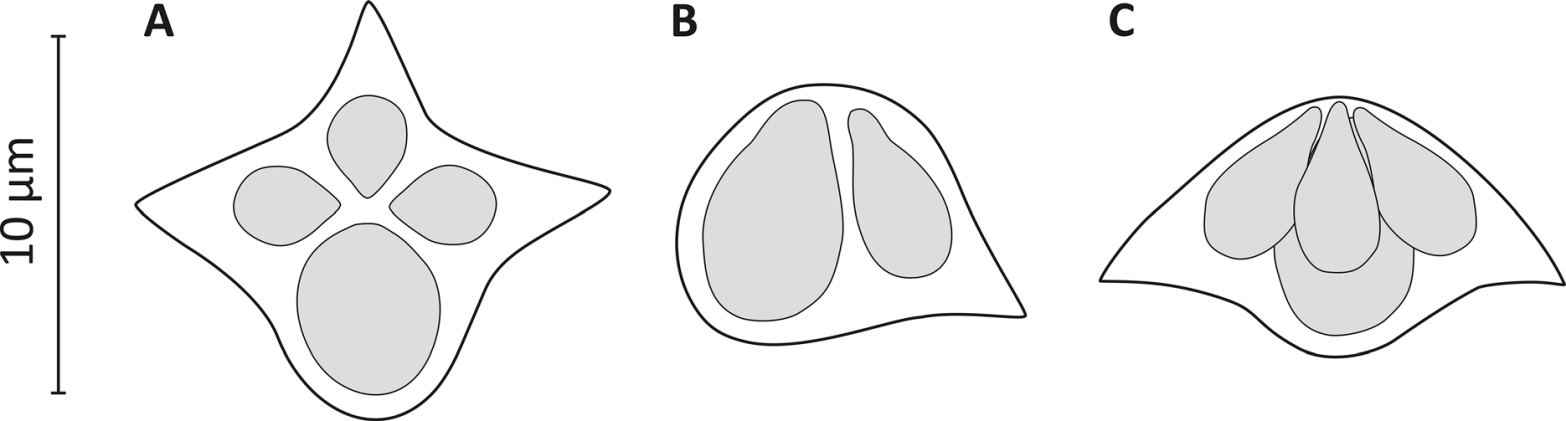
Illustrations of *Kudoa encrasicoli* **n. sp.** myxospores in apical (A) and lateral (B, C) views

*Description* (Figs. 1A-1G, and 2; Table 2)

**Table 2:** Myxospore measurements (in µm) and morphometric ratios of *Kudoa encrasicoli* **n. sp.** and *K. thyrsites* ex *Sardina pilchardus.* Thirty measurements were taken for each character and species. Data are mean ± standard deviation with range in parentheses. W1: myxospore width considering the SVs bearing the large (A) and the small (C) opposite PC; W2: myxospore width considering the two “intermediate” (B-D) PC-bearing SVs; T: myxospore thickness; L: myxospore length; AL: length of PC A; AW: width of PC A; BL: length of PC B; BW: width of PC B; CL: length of PC C; CW: width of PC C; DL: length of PC D; DW: width of PC D; B-DW: combined widths of PCs B and D; B-C-DW: combined width of PCs B, C and D; B-C-DL: combined length of polar capsules B, C and D; AL/AW: length/width ratio for PC A; BL/BW: length/width ratio for PC B; CL/CW: length/width ratio for PC C; DL/DW: length/width ratio for PC D; B-C-DL/B-C-DW: combined length/width ratio for the PCs B, C and D; AL/CL: ratio between the lengths of PCs A and C; AW/CW: ratio between the widths of PCs A and C

Measurement/ratio	*K. encrasicoli* **n. sp.**	*K. thyrsites* ex *S. pilchardus*
W1^1^	10.8 ± 0.7 (9.1–12.3)	15.2 ± 0.9 (13.4–16.8)
W2^1^	11.3 ± 0.9 (9.5–13.4)	16.2 ± 0.9 (14.1–17.9)
T^1^	6.7 ± 0.4 (5.8–7.4)	7.1 ± 0.4 (6.4–8.1)
L^1^	6.9 ± 0.5 (5.8–7.5)	6.6 ± 0.5 (5.8–7.7)
AL_a_^1^	4.8 ± 0.3 (4.2–5.6)	4.1 ± 0.3 (3.6–5.2)
AL_l_^1^	6.8 ± 0.4 (5.9–7.6)	6.5 ± 0.3 (6–7.3)
AW_a_^1^	4.1 ± 0.2 (3.6–4.4)	3.4 ± 0.2 (2.9–3.7)
CL_l_^1^	5.0 ± 0.3 (4.4–5.4)	5.3 ± 0.3 (4.4–5.8)
CW_a_^1^	2.4 ± 0.2 (2.0–3.0)	2.4 ± 0.2 (1.9–2.7)
B-DW_a_^1^	–	2.6 ± 0.2 (2.2–3.2)
B-C-DW_a_^1^	2.4 ± 0.2 (2.0–3.0)	–
AL^2^	6.3 ± 0.2 (5.9–6.8)	6.2 ± 0.3 (5.8–6.7)
AW^2^	4.3 ± 0.2 (3.9–4.6)	3.4 ± 0.2 (3.1–3.8)
BL^2^	4.4 ± 0.2 (4.0–4.8)	4.9 ± 0.3 (4.4–5.4)
BW^2^	2.1 ± 0.1 (1.9–2.4)	2.6 ± 0.1 (2.4–2.9)
CL^2^	4.3 ± 0.2 (3.5–4.6)	4.1 ± 0.4 (2.7–4.7)
CW^2^	2.1 ± 0.1 (1.8–2.4)	2.3 ± 0.1 (2.2–2.6)
DL^2^	4.4 ± 0.2 (3.9–4.7)	4.9 ± 0.3 (4.3–5.4)
DW^2^	2.1 ± 0.1 (1.8–2.5)	2.6 ± 0.1 (2.4–2.8)
B-C-DL^2^	4.4 ± 0.2 (3.5–4.8)	–
B-C-DW^2^	2.1 ± 0.1 (1.8–2.5)	–
AL/AW^2^	1.49 ± 0.09 (1.33–1.63)	1.83 ± 0.07 (1.69–1.94)
BL/BW^2^	2.11 ± 0.14 (1.88–2.42)	1.92 ± 0.08 (1.72–2.07)
CL/CW^2^	2.07 ± 0.16 (1.73–2.48)	1.77 ± 0.14 (1.25–2.00)
DL/DW^2^	2.10 ± 0.15 (1.90–2.37)	1.90 ± 0.12 (1.31–2.23)
B-C-DL/B-C-DLW^2^	2.10 ± 0.10 (1.90–2.30)	–
AL/CL^2^	1.48 ± 0.10 (1.31–1.73)	1.52 ± 0.16 (1.31–2.23)
AW/CW^2^	2.05 ± 0.13 (1.82–2.28)	1.46 ± 0.10 (1.26–1.67)

^1^Measurements taken on intact myxospores according to the guidelines given by Burger and Adlard (2010) for stellate myxospores with one PC larger than the others (a: measurement taken on myxospores in apical view; l: measurement taken on myxospores in lateral view).

^2^Measurements (or ratios calculated from measurements) taken on flattened and immobilized myxospores (PCs are thus positioned in a perfect longitudinal view).

Elongated but not visible macroscopically polysporic plasmodia (Figs. 1A and 1B), often with blunt ends, measuring 455 ± 367.3 (130-980) µm in length and 98.7 ± 22.3 (80-140) µm in width. Plasmodia located between the myofibers of trunk muscles, with synchronous development and no host reaction around them. Mature myxospores almost “stellate” in apical view (Figs. 1C, 1E, 2A) with three thin-walled SVs exhibiting pointed edges (sometimes with slightly invaginated vertices) and bearing pyriform PCs equal in size, and one SV with a rounded (rarely bluntly pointed) edge bearing a large PC. In lateral view (Figs. 1D, 1F, 2B), myxospores are slightly scalene pyramidal with one rounded edge corresponding to the large PC-bearing SV. Turns of the polar filament are not discernible. Intact myxospores (n=30; measurements in µm) measure 10.8 ± 0.7 (9.1-12.3) in width 1 (W1), 11.3 ± 0.9 (9.5-13.4) in width 2 (W2), 6.7 ± 0.4 (5.8-7.4) in thickness (T), 6.9 ± 0.5 (5.8-7.5) in length (L). Large PC is 6.8 ± 0.4 (5.9-7.6) long (AL_l_) and 4.1 ± 0.2 (3.6-4.4) wide (AW_a_); small PCs are 5.0 ± 0.3 (4.4-5.4) long (CL_l_) and 2.4 ± 0.2 (2.0-3.0) wide (B-C-DW_a_). Large and small PCs in flattened myxospores are 6.3 ± 0.2 (5.9-6.8) long (AL) and 4.3 ± 0.2 (3.9-4. 6) wide (AW), and 4.4 ± 0.2 (3.5-4.8) long (B-C-DL) and 2.1 ± 0.1 (1.8-2.5) wide (B-C-DW), respectively. The length/width ratios for the large PC (AL/AW) and for the 3 small PCs (B-C-DL/B-C-DW; data combined) are 1.49 ± 0.09 (1.33-1.63) and 2.10 ± 0.10 (1.90-2.30), respectively. The ratio between the lengths of the large PC and the opposite small PC (AL/CL) is 1.48 ± 0.10 (1.31-1.73) while the ratio between the widths of the large PC and the opposite small PC (AW/CW) is 2.05 ± 0.13 (1.82-2.28).

### Remarks

Considering all skeletal muscle-dwelling kudoids having stellate myxospores in apical view and 4 PCs, the species *K. bengalensis* Sarkar & Mazumder, 1983*, K. cynoglossi* Obiekezie & Lick, 1994*, K. haridasae* Sarkar & Ghosh, 1991*, K. kabatai* Kovaleva Shulman & Yakovlev, 1979*, K. lunata* Lom, Dyková & Lhotákova, 1983*, K. miniauriculata* Whitaker, Kent & Sakanari, 1996*, K. ogawai* Yokoyama, Yanagida & Shirakashi, 2012*, K. quadricornis* Whipps, Adlard, Bryant & Kent, 2003*,* and *K. ramsayi* Kalavati, Brickle & MacKenzie, 2000 can be easily differentiated from *K. encrasicoli* **n. sp.** since all of them have myxospores with 4 PCs equal or almost equal in size (morphological characteristics reviewed by Moran et al., 1999 and Eiras et al., 2014). Something similar occurs in the case of *K. empressmichikoae* Kasai, Setsuda & Sato, 2017*,* the myxospores of which have furthermore a spherical body with posterolateral SV projections (Kasai et al., 2017). Regarding species with myxospores exhibiting 4 PCs unequal in size, *K. megacapsula* Yokoyama & Itoh, 2005 and *K. unicapsula* Yurakhno, Ovcharenko, Holzer, Sarabeev & Balbuena, 2007 are different from *K. encrasicoli* **n. sp.** because their three small PCs are few developed, vestigial, or even degenerated (Yokoyama & Itoh, 2005; Yurakhno et al., 2007). In addition, myxospores of the latter species are rectangular in apical view and their SVs present curved edges.

**Fig. 3:**
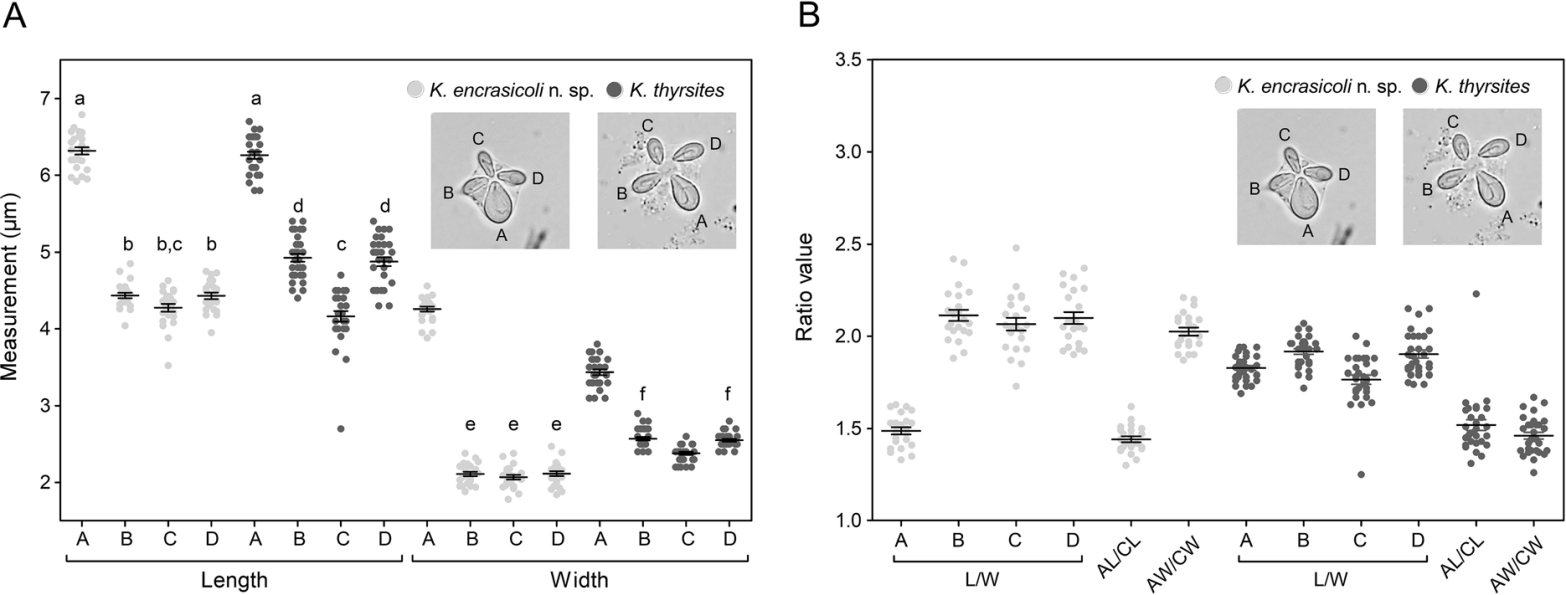
A. Comparative scatter plot showing the individual measurements (length and width, in µm) and the mean ± standard error obtained for the four PCs (A-D in photographs) of flattened myxospores of *Kudoa encrasicoli* **n. sp.** (n= 30; light grey) and *K. thyrsites* from *Sardina pilchardus* (n= 30; dark grey). Values of length or width identified with the same letter did not show significant differences in a One-way ANOVA followed by a Tukey’s multiple comparison test. All other comparisons between lengths or widths were significantly different (p < 0.05). B. Comparative scatter plot showing the individual values and the mean ± standard error of the ratios between the length and the width (L/W) of the four PCs (A-D in photographs), the lengths of the PCs A and C (AL/CL), and the widths of the PCs A and C (AW/CW). Ratios were calculated from the measurements taken from flattened myxospores of both species. Interspecies comparison of each ratio by using an unpaired t test revealed high significant differences (p < 0.0001) in all cases excepting the AL/CL ratio (p= 0.0430)

**Fig. 4:**
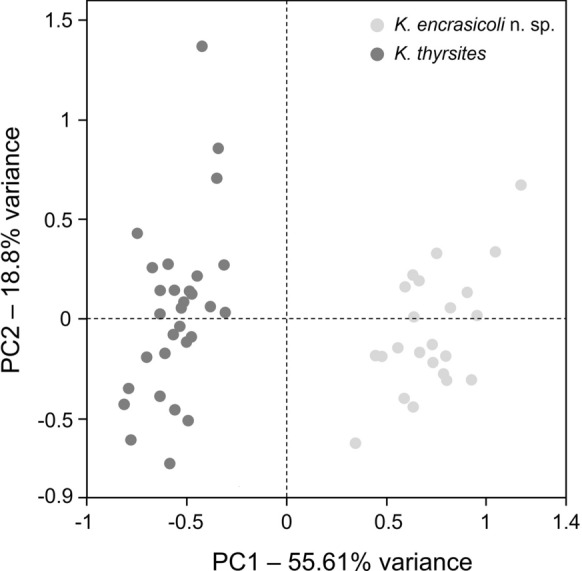
Principal component analysis based of 8 morphometric variables (lengths and widths of the four PCs taken from flattened myxospores) of *Kudoa encrasicoli* **n. sp.** (light grey circles) and *K. thyrsites* ex *Sardina pilchardus* (dark grey circles). Percentages of the total variance explained by the principal component 1 (PC1) and the principal component 2 (PC2) are indicated in their respective axis titles

The characteristic rounded (rarely bluntly pointed) edge of the large PC-bearing SV of *K. encrasicoli* **n. sp.** myxospores allows to distinguish this new species from *K. akihitoi* Kasai, Setsuda & Sato, 2017, *K. cheilodipteri* Heiniger, Cribb & Adlard, 2013, *K. cruciformum* (Matsumoto, 1954), *K. gunterae* Burger & Adlard, 2010, *K. lateolabracis* Yokoyama, Whipps, Kent, Mizuno & Kawakami, 2004, *K. minithyrsites* Whipps, Adlard, Bryant, Lester, Findlay & Kent, 2003, *K. mirabilis* Naidenova & Gaevskaya, 1991, *K. parathyrsites* Kasai, Li, Mafie & Sato, 2016*, K. thyrsites* (=*K. histolytica*; see myxospores from *S. pilchardus* in Figs. 1H-1K), and *K. whippsi* Burger & Adlard, 2010 which are tipically stellate in form because of the clearly pointed edges of the four SVs (Whipps et al., 2003a; Yokoyama et al., 2004; Whipps & Kent 2006; Burger & Adlard 2010; Heiniger et al., 2013; Eiras et al., 2014; Kasai et al., 2016b, 2017), and from *K. valamugili* Kalavati & Anuradha, 1993 which has quadrate myxospores in apical view and SVs with curved edges (morphology reviewed by Eiras et al., 2014). Concerning *K. thyrsites*, it should be also stressed that i) its plasmodia exhibit intrafibrillar development, ii) its myxospores are widest, and iii) the PC C located opposite to the large PC A is significantly smaller than the two intermediate PCs B and D (Figs. 1I-1K, 3A). Plasmodia of *K. encrasicoli* **n. sp.**, by contrast, are located interfibrillarly while the three small PCs B, C and D are equal in size, as we have demonstrated statistically in this work (Fig. 3A). Although a 1.8-1.9 AL/CL ratio has been recently reported for *K. thyrsites* by Giulietti et al. (2019), that value contrasts with the equivalent ratio calculated by us for the *K. thyrsites* myxospores obtained from *S. pilchardus* (1.52 ± 0.16) and from other fish hosts by other authors (in all cases ≤ 1.58; Whipps & Kent 2006; Kasai et al., 2016b). While the AL/CL ratio of *K. thyrsites* ex *S. pilchardus* was slightly although significantly higher to that of *K. encrasicoli* **n. sp.** (1.48 ± 0.10) the AW/CW ratio calculated for *K. thrysites* ex *S. pilchardus* (1.46 ± 0.10) was clearly and significantly lower than that obtained for *K. encrasicoli* **n. sp.** (2.05 ± 0.13) (Fig. 3B). In addition, the AL/AW ratio of the new species described herein was significantly lower than that of *K. thyrsites* ex *S. pilchardus* (1.49 ± 0.09 vs. 1.83 ± 0.07; Fig. 3B). These results confirm that the large PC A of *K. encrasicoli* **n. sp.** is clearly widest than that of *K. thyrsites* as it can be observed in Figures 1E-1G and 1I-1K. These findings were also supported by the PCA (Fig. 4) based on the lengths and widths of the 4 PCs (8 variables) taken on flattened myxospores of both species. Thus, the principal component 1 (PC1), in which the large PC A width followed by the lengths and widths of small PCs B and D (both contiguous to the large PC) accounted for the highest weight, explained 55.61% of total variation and clearly differentiated both species in two separate clusters.

Finally, although most of kudoid species with muscular tropism, including *K. thyrsites*, develop their plasmodia within the muscle fibers, those of *K. kabatai* (morphologic myxospore differences already discussed above), *K. azoni* Aseeva, 2004*, K. crumena* Iversen & van Meter, 1967*, K. iwatai* Egusa & Shiomitsu, 1983*, K. kenti* Burger & Adlard, 2010 and *K. yasai* Araújo-Neto, da Silva, Hamoy, Matos & Abrunhosa, 2020 have been reported, as *K. encrasicoli* **n. sp.**, in interfibrilar location (Burger & Adlard 2010; Eiras et al., 2014; Cardim et al., 2020). The last five species, however, have quadrangular (not stellate) myxospores, SVs with curved edges, and PCs equal or very similar in size.

Pairwise genetic divergence (*p*-distance) between the VKE isolate and the VKS and KPT isolates was 1.1% and 3.8% for the 18S rDNA and 28S rDNA respectively. The 18S rDNA sequences of isolate VKS is identical to the isolate KPT and differs only by 0.1% for the 28S rDNA sequences (Table S1).

The genetic distance between the *K. thyrsites* 18S rDNA sequences available from GenBank and the VKE isolate was 0.6% for the blue whitting host (*Micromesistius poutassou*), and 0.8% for the silver scabbardfish host (*Lepidopus caudatus*) from the Portuguese and Spanish coasts, respectively, followed by DNA sequences from hosts of the Pacific Ocean (Canada) with a genetic distance of 1.0%. The remaining sequences from *K. thyrsites* range from 1.1 to 1.5%, and the most distant are sequences from hosts originating in Japan (2.4%). It should be noted that the genetic distance between VKE and the *K. thyrsites* sequence (AY078430) obtained from its type host (*Thyrsites atun*) in South Africa is 1.2%. For 28S rDNA, the genetic distance between the VKE isolate and the remaining *K. thyrsites* sequences range from 3.8 to 9.3%. The closest sequences are from Atlantic and Pacific Ocean hosts (3.8%), and the most distant are from Japanese hosts (9.3%). For *K. thyrsites* ex *T. atun* the distance is 3.8%.

Comparing the genetic distance between *K. thyrsites* sequence from the type host to other *K. thyrsites* described from fishes of the Atlantic Ocean (inclundig the isolates VKS and KPT) there were only a small variation 0.0-0.1% for 18S and 0.0-0.3% for the 28S rDNA. Calculating the genetic distance of *K. thyrsites* from the type host to sequences of *K. thyrsites* from other geographic regions, the minimal and maximum variation are much larger for the 18S and 28S rDNA, respectively: 0.5-0.6% and 1.6-1.7% for species from the American Pacific coast (Canada, U.S.A., and Chile), 0.6-0.7% and 2.2% for Australian coast, and 0.9-1.9% and 7.8% for Indo-Pacific region (Japan, Tanzania and South Africa).

**Fig. 5:**
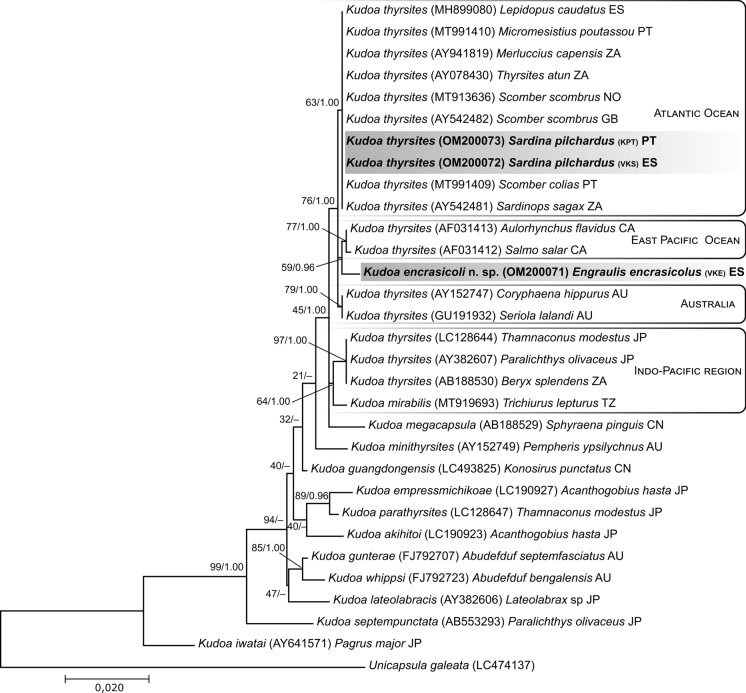
Maximum likelihood phylogenetic tree of the small subunit ribosomal DNA sequences of *Kudoa* species from the *K. thyrsites* complex and other selected sequences. Nodes values represent nodal support by bootstrap for 1000 replicates and Bayesian inference posterior probabilities from 1,000,000 generations. New sequences from this study are in bold. GenBank accession numbers are given inside parentheses, followed by host name and geographic location (country abbreviation codes ISO 3166-1)

**Fig. 6:**
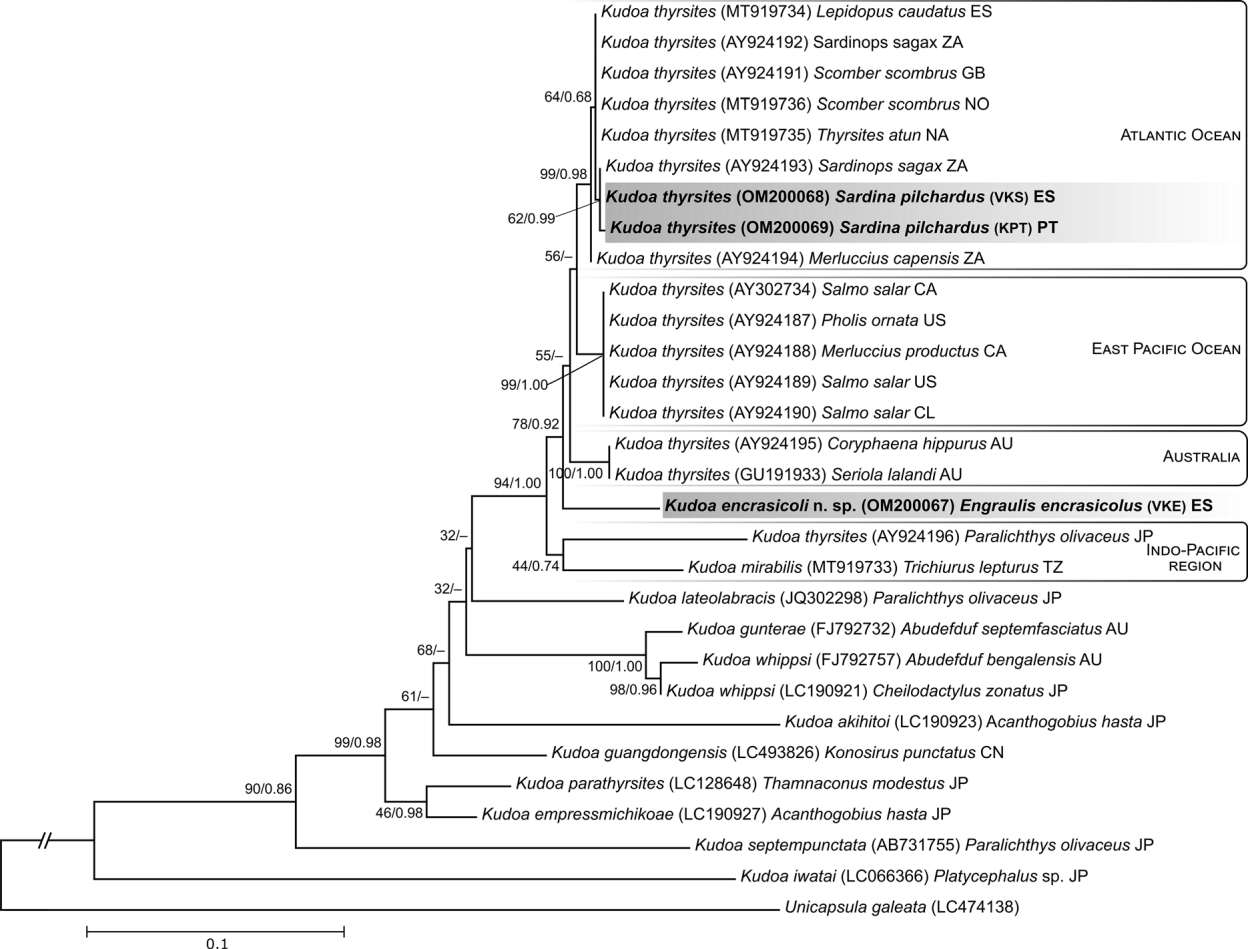
Maximum likelihood phylogenetic tree of the large subunit ribosomal DNA sequences of *Kudoa* species from the *K. thyrsites* complex and other selected sequences. Nodes values represent nodal support by bootstrap for 1000 replicates and Bayesian inference posterior probabilities from 1,000,000 generations. New sequences from this study are in bold. GenBank accession numbers are given inside parentheses, followed by host name and geographic location (country abbreviation codes ISO 3166-1)

In both phylogenetic trees, for the 18S and 28S rDNA genes, the DNA sequences assigned to the species *K. thyrsites* are equally distributed according to the geographic regions where their hosts were collected. However, the DNA sequence of *K. encrasicoli* **n. sp.** does not have a coherent position in the different trees. In the phylogenetic tree of 18S rDNA sequences, *K. encrasicoli* **n. sp.** appears clustering with sequences of species collected from fishes of the Pacific Ocean region (Canada and British Columbia), this clustering occurs when both ML and BI methods are used (Fig. 5). However, this clustering does not occur in the phylogenetic tree with 28S rDNA sequences, using the ML and BI methods (Fig. 6). In these trees *K. encrasicoli* **n. sp.** appears isolated either in a more basal position in relation to the clades of the Atlantic Ocean, the Pacific Ocean and Australia (ML), or as a sister taxa of each of these clusters (BI). The only consistency across all trees and methods is the basal position of *K. thyrsites* species from the Indo-Pacific region (Japan, Tanzania and South Africa), together with *K. mirabilis*, in relation to *K. thyrsites* from other geographic regions. Exceptionally, *K. thyrsites* (AB188530) from *Beryx splendens* from South Africa cluster with species from the Indo-Pacific region, possibly due to the fishing zone since South Africa is bordered to the west by the Atlantic Ocean and to the east by the Indian Ocean.

## Discussion

Our results clearly demonstrate that *K. encrasicoli* **n. sp.** differs from *K. thyrsites* in i) morphological characters (rounded- vs. pointed-edged large PC-bearing SV), ii) morphometrical data (W1, W2, BL, DL, AW, BW, CW, and DW mean values as well as all mean ratios, but especially AW/CW, were clearly different in both species), and iii) microhabitat preferences (intra- vs. interfibrillar). As far as we know only *K. thyrsites* has been reported to date infecting the skeletal muscle of engraulids, specifically in the species *E. australis* (12% prevalence) and *E. japonicus* (50% prevalence) from Australian and Japanese waters, respectively (Langdon et al., 1992). Although no morphological and morphometrical details of myxospores were given and no molecular analysis was carried out, the visual examination of the photographs presented by the authors suggest that myxospores found in both engraulids could be similar in shape to those of *E. encrasicoli,* also contrasting with the typically stellate myxospores of *K. thyrsites* detected by the authors in the Australian clupeid *Sardinops sagax neopilchardus*. Similarly to our observations, no myoliquefactive changes, or only focal softening (in *E. japonicus*), were observed by Langdon et al. (1992) in infected engraulids, with severe myoliquefaction being reported in the clupeid *S. sagax neopilchardus*. New studies including morphological, morphometrical and molecular approaches will be therefore needed to know if *K. encrasicoli* **n. sp.**, or very similar species, are able to develop in the skeletal muscle of other engraulid species.

Certain morphological characteristics of myxospores have been traditionally used for describing new species belonging to the genus *Kudoa.* However*,* the use of heterogenous and incomplete criteria for measuring myxospores have considerably hindered the comparison between new and described species. In this sense, some authors have proposed useful guidelines as well as new features to produce more accurate and homogeneous descriptions of myxospores belonging to the genus *Kudoa* (Lom & Arthur, 1989; Langdon, 1991; Whipps & Kent, 2006; Burger & Adlard,^2010^; Giulietti et al., 2019)*.* Despite the obvious utility of these recommendations, some technical difficulties remain unresolved. Thus, as it has been also stressed by Giulietti et al. (2019), the small size and the three-dimensional and complex morphology of kudoid myxospores as well as the unwanted movements (sometimes rotatory) of these forms in the wet mounts used for measuring hinder to obtain accurate measurements, particularly in the case of PCs, the dimensions of which can be seriously conditioned by the spatial orientation of myxospore. Thus, in stellate myxospores with unequal PCs in which the lengths of the large and small opposite PCs should be taken on myxospores disposed in lateral view according to Burger & Adlard (2010), the measurements obtained can be largely dependent on the inclination of the longitudinal axis of the myxospore. Furthermore, the anterior and, in a lesser extent, the posterior margins of PCs are not easily delimited (also stressed by Burger & Adlard, 2010) because of their pyramidal disposition and refringence thus conditioning an accurate measuring of their dimensions. These aspects, coupled with the past absence of consensual guidelines for measuring myxospores, are probably responsible for the enormous variability observed in the measurements given for certain apparently generalist species such as *K. thyrsites* which have been the subject of numerous descriptions from different host species (Langdon, 1991; Yokoyama et al., 2004; Whipps & Kent, 2006; Kasai et al., 2016b; Giulietti et al., 2019). In this regard, we have observed that in stellate or almost stellate myxospores containing 4 SVs/PCs (i. e. *K. thyrsites* and *K. encrasicoli* **n. sp.**) the length and width of PCs are more easily and accurately measured when myxospores become immobilized and slightly flattened as a result of the slight pressure exerted by the coverslip as saline evaporates during the microscopic examination. In addition, other morphological characteristics as PC shape and polar filament coils can also be more easily visualized in flattened myxospores helping to generate more complete descriptions. This methodology has been already used by Kasai et al. (2016b) to visually demonstrate the different dimensions of the four PCs in *K. thyrsites.* In our case, this approach allowed a reliable morphometrical comparison between *K. encrasicoli* **n. sp.** and the species *K. thyrsites* (ex *S. pilchardus*) demonstrating that the lengths of the small PCs contiguous to the large PC, and especially the large PC width, were not only significantly different in both species but also particularly useful for their PCA discrimination. These results contradict Giulietti's arguments stating that the width of PCs is not a relevant character for describing and differentiating *Kudoa* species (Giulietti et al., 2019). In fact, largest width of the large PC of *K. encrasicoli* **n. sp.** is a clear and robust morphometrical difference with *K. thyrsites* and it is probably the cause of the characteristic rounded edge of the large PC-bearing SV and the almost stellate appearance of *K. encrasicoli* **n. sp.** myxospores*.* On the other hand, in agreement with Giulietti et al. (2019), the allometric ratios calculated from the PC measurements taken on flattened myxospores, and their statistical comparison, were especially useful for differentiating *K. encrasicoli* **n. sp.** and *K. thyrsites.* For all these reasons, the measurements of PCs taken on flattened myxospores should be added to descriptions of new species having stellate myxospores with 4 SVs/PCs.

The genetic divergence between the DNA sequences for the 18S and 28S rDNA attributed to the species *K. thyrsites* from various host species in the Atlantic Ocean had a variation from 0 to 0.1% and 0 to 0.3% respectively, while for the other geographic regions the minimum divergence is 0.5% and 1.6% for the eastern Pacific Ocean and a maximum of 1.9% and 7.8% for species from the Indo-Pacific region. So, for the Atlantic Ocean, comparing the sequences of the isolate VKE from the host *E. encrasicolus* to *K. thyrsites* from the type host the variation is much larger (1.2% and 3.8%, respectively). This reinforces the morphological arguments for the description of a new species, *K. encrasicoli* **n. sp.**

The clustering of *K. thyrsites* species according to the geographic regions described in the bibliography (Giulietti et al., 2020; Cavaleiro et al., 2021) and observed in the phylogenetic trees in this study is disturbed by the presence of *K. encrasicoli* **n. sp.** in a position away from the Atlantic Ocean cluster, although its phylogenetic position is still unstable. These results reinforce the previous studies that suggest that *K. thyrsites* is a complex of close but distinct species, i.e. cryptic species (Whipps & Kent, 2006; Giulietti et al., 2020), as already suggested by the 'intrusion' of the species *K. mirabilis* in the *K. thyrsites* cluster of the Indo-Pacific region. On the other hand, the description of this new species in a host from the North Atlantic Ocean, but which does not fit into the typical cluster of *K. thyrsites* from the Atlantic Ocean also reveals how artificial this geographic division of the *K. thyrsites* DNA sequences is. It becomes evident the need to make a review of this group of *Kudoa* species and the expansion of the sampling to the various geographic regions and hosts.

## Supplementary Information

Below is the link to the electronic supplementary material.

Supplementary file1 (XLSX 23 kb) Table S1. pDistance1.xlxs. Table S1 legend. Spreadsheet with the p-Distance tables for small and large subunit ribosomal DNA sequences of Kudoa species, calculated by MEGA v7 software, using the same aligned sequences matrix used for the phylogenetic analysis.

## Data Availability

All data not included in manuscript will be made available upon reasonable request.
